# Native Whey Induces Similar Post Exercise Muscle Anabolic Responses as Regular Whey, Despite Greater Leucinemia, in Elderly Individuals

**DOI:** 10.1007/s12603-018-1105-6

**Published:** 2018-09-18

**Authors:** Håvard Hamarsland, S. N. Aas, A. L. Nordengen, K. Holte, I. Garthe, G. Paulsen, M. Cotter, E. Børsheim, H. B. Benestad, T. Raastad

**Affiliations:** 1 Department of Physical Performance, Norwegian School of Sport Sciences, P.O. Box 4014, Ullevål Stadion, 0806 Oslo, Norway; 2 Norwegian Olympic Federation, Oslo, Norway; 3 Department of Nutrition, Institute of Basic Medical Sciences, University of Oslo, P.O. Box 1046, 0317 Blindern, Oslo, Norway; 4 Arkansas Children’s Nutrition Center, Little Rock, AR USA; 5 Arkansas Children’s Research Institute, Little Rock, AR USA; 6 Departments of Pediatrics and Geriatrics, University of Arkansas for Medical Sciences, Little Rock, AR USA; 7 Section of Anatomy, Institute of Basis Medical Sciences, University of Oslo, Oslo, Norway

**Keywords:** Skeletal muscle, supplementation, amino acids, protein quality, stable isotopes

## Abstract

**Objective:**

Elderly muscle seems less sensitive to the anabolic stimulus of a meal. Changes in blood concentrations of leucine are suggested as one important trigger of the anabolic response in muscle. The aim of this study was to investigate whether native whey protein, containing high amounts of leucine, may be a more potent stimulator of muscle protein synthesis (MPS) in elderly than regular whey protein (WPC-80) or milk.

**Design:**

Randomized controlled partial crossover.

**Setting:**

Norwegian School of Sport Sciences.

**Participants:**

21 healthy elderly men and women (≥70 years).

**Intervention:**

Participants received either 20 g of WPC-80 and native whey (n = 11) on separate days in a crossover design, or milk (n = 10). Supplements were ingested immediately and two hours after a bout of lower body heavy-load resistance exercise.

**Measurements:**

Blood samples and muscle biopsies were collected to measure blood concentrations of amino acids by gas-chromatography mass spectrometry (GCMS), phosphorylation of p70S6K, 4E-BP1 and eEF-2 by immunoblotting and mixed muscle fractional synthetic rate (FSR) by use of [2H5]phenylalanine-infusion, GCMS and isotope-ratio mass spectrometry.

**Results:**

Native whey increased blood leucine concentrations more than WPC-80 (P < 0.05), but not p70S6K phosphorylation or mixed muscle FSR. Both whey supplements increased blood leucine concentrations (P < 0.01) and P70S6K phosphorylation more than milk (P = 0.014). Native whey reached higher mixed muscle FSR values than milk (P = 0.026) 1-3h after exercise.

**Conclusions:**

Despite greater increases in blood leucine concentrations than WPC-80 and milk, native whey was only superior to milk concerning increases in MPS and phosphorylation of P70S6K during a 5-hour post-exercise period in elderly individuals.

**Electronic Supplementary Material:**

Supplementary material is available for this article at 10.1007/s12603-018-1105-6 and is accessible for authorized users.

## Introduction

Advancing age is accompanied by loss of muscle mass and strength. This condition can proceed to sarcopenia, which is linked to loss of independent living (1) and several comorbidities (2). The need to understand the causal mechanisms of loss, and effects of interventions to counteract sarcopenia increases with an increased aged population (3). The loss of muscle mass must result from an imbalance between muscle protein synthesis (MPS) and muscle protein breakdown (MPB). Because fasting MPS has been shown not to differ between young and old (4), focus has been on the response to the anabolic effect of protein intake and resistance exercise in elderly. Several, but far from all, studies have reported a reduced MPS response to protein ingestion and resistance exercise in elderly compared to young, termed anabolic resistance (5). Although the optimal post exercise protein intake in order to maximally stimulate MPS remains to be identified it seems to be somewhere between 20 and 40 g of high quality protein in young (6) and increased with advancing age (7). Reaching this higher protein intake may become a challenge as appetite is often depressed in elderly (8). As a consequence, quality and the ability of a protein source to stimulate MPS become more important in the elderly population. The anabolic effect observed after protein intake is driven by the essential amino acids (EAA; (9) and especially leucine (10)). Adding leucine to a suboptimal protein dose can overcome the anabolic resistance and stimulate MPS in elderly to the same levels as in young (11, 12). Thus, a protein with higher levels of leucine may allow for a greater stimulation of MPS when the protein dose is suboptimal.

Native whey protein is produced by filtration of unprocessed raw milk. This production method leaves proteins intact and results in a higher leucine content than other high quality proteins such as regular whey (WPC-80) and bovine milk. WPC-80 is a sub-product of cheese production, during which it is chemically treated and heated. WPC-80 is the most common protein used in protein supplements and is often regarded as the gold standard of protein sources. The higher leucine content may give native whey a greater MPS-stimulating ability than WPC-80. In addition, as chemical and heat-treatment can render some amino acids unavailable for utilization in the body (13) the more gentle process of filtration may alter the physiological effects of native whey compared to WPC-80 and milk. Earlier studies have shown beneficial effects of whey protein on health outcomes (14) and soluble (native) whey on muscular function in elderly (15). We have previously shown native whey to induce greater leucine blood concentrations than WPC-80 and milk, in young participants after resistance exercise (16). Accordingly, we hypothesize native whey would be a more potent stimulator of MPS than WPC-80 and milk in elderly. The observation that studies combining protein supplementation and resistance exercise report less anabolic resistance in elderly than either intervention alone suggests this combination to be a promising approach in preventing sarcopenia in elderly (5). We therefore combined the protein intake with a bout of resistance exercise. This study included elderly over the age of 70 years. As the loss of muscle mass and strength is generally considered to accelerate after the age of 70 years (17, 18), interventions like the present may be most effective in this age group.

The main objective of this study was to compare the effects of post exercise supplementation of WPC-80 or native whey on the acute (1-5 hours) MPS-response of elderly participants. In addition, as a readily available high quality food source of protein may be more preferable than supplements, we also compared the whey proteins with similar amounts of bovine milk (1% fat).

## Materials and methods

### Participants and ethical approval

Twenty-two healthy, recreationally active elderly (+70 yrs) men and women were included in the study (Table 1). 11 participants reported to be engaged in resistance exercise for an average 3 hours per week. These participants were equally distributed between groups. All except one participant reported recreational walking for an average of 2.5 hours per week. One participant withdrew from the study due to illness before the start of the experiment. All participants underwent a medical screening before entering the study. To take part, participants had to be healthy and without any injuries to the musculoskeletal system that could interfere with the exercise. Individuals with lactose intolerance, milk allergy or using any dietary supplements were excluded. Participants were nonsmokers with no cardiovascular disease. Two took statins and three took medication for high blood pressure. The study was approved by the Regional Ethics Committee for Medical and Health Research of South-East Norway (2014/834/REK sørøst C) and performed in accordance with the Declaration of Helsinki. All participants signed a written informed consent form before entering the study. The trial was registered at clinicaltrials.gov as NCT03033953.

**Table 1:** Participant characteristics

**Characteristics**	**Milk**	**Whey**	**P values for group differences**
N (♂/♀)	(7/3)	(6/5)	
Age (years)	75 ± 4	73 ± 3	0.057
Body mass (kg)	76.3 ± 17.8	70.0 ± 11.6	0.487
Lean body mass (kg)	52.8 ± 11.4	50.5 ± 10.5	0.638
Body fat (%)	27.7 ± 7.2	27.4 ± 7.1	0.937
Leg press 8 RM (kg)	148 ± 67	134 ± 51	0.749
Knee extensions 8 RM (kg)	62 ± 22	57 ± 19	0.740
Total weight lifted (kg)	6572 ± 2652	6134 ± 2024 / 6128 ± 1971	0.683

**Figure 1:**
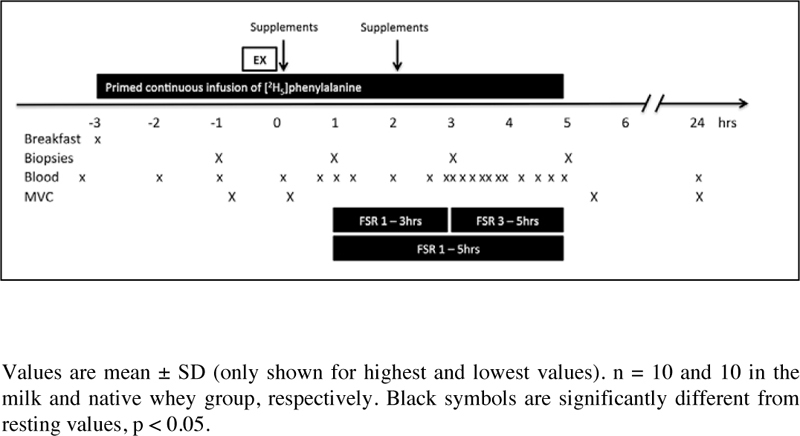
Experimental design

### Study design

**Figure 2:**
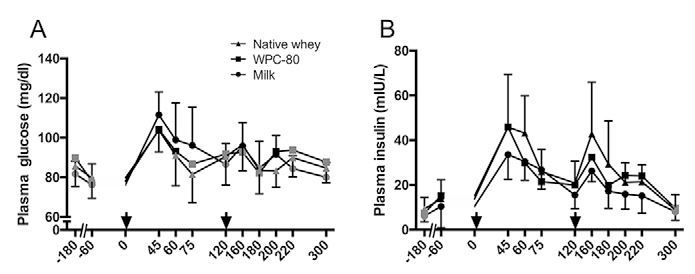
Blood concentrations of serum glucose (A), insulin (B) following intake of 20 g milk protein, WPC-80 or native whey immediately after a bout of heavy leg resistance exercise. Arrow indicates time point of protein supplement ingestion

This study was a double blinded, randomized, partial crossover, controlled trial (Figure 2). Each participant was randomly assigned to one of two groups. The milk group did the protocol once, whereas the whey group was exposed to the protocol two times, once consuming WPC-80 and once consuming native whey, in a randomized order. The partial crossover was chosen in order to have a stronger paired comparison between WPC-80 and native whey. Participants receiving WPC-80 or native whey had an average of 8 ± 1.4 and 9 ± 1.6 days between experiments, respectively. After a standardized breakfast participants performed a bout of highload leg-resistance exercise before ingesting a drink containing 20 g of protein from milk, WPC-80 or native whey, both immediately after and again 2 hours after exercise. The reason for giving two servings of supplements was to avoid the large energy deficiency potentially occurring during the later part of the 5-hour measurement period. Giving one 40 g serving of supplement may have been enough to maximally stimulate MPS with all supplements and thus disguise the effect of a higher quality of the whey supplements. Blood samples were collected to measure concentrations of amino acids, glucose, insulin, urea and creatine kinase (CK). MPS and related intracellular signaling were measured during a 5-hour recovery period combining biopsies and tracer infusion of [2H5] phenylalanine. In addition we measured recovery of muscle force-generating capacity by maximal isometric voluntary contractions (MVC) prior to, 10 min, 5.5 and 24 hours after exercise. The 24-hour time point was chosen as we expected the participants to not be fully recovered by this time and possible differences between groups to be evident. In addition time of day variations in performance would not affect our results.

### Familiarization

During the four weeks prior to the study, participants met six times in the laboratory for supervised establishment of their 4 sets of 8RM of bilateral leg press and knee extension, and to familiarize to the standardized workout and the MVC-test. All participants were asked to refrain from physical exercise for 48 hours prior to the experiments.

### Diet

Participants completed two 24-hour dietary recall interviews. A trained dietitian conducted the recall interviews and analyzed dietary nutrient content using the software Mat på Data 5.1 (Mattilsynet, Oslo, Norway, 2009). The standardized breakfast was oatmeal with water, sugar and rapeseed oil, containing 25 kJ, 0.11 g of protein, 0.3 g of fat and 0.7 g of carbohydrates per kg body mass. Participants were provided with a written individual diet plan (30 kcal/kg and 1.3 g protein/kg per day) and pre-packaged food for the day before the experiment, and for the rest of the experimental period (2.5 days with standardized diet in total). Participants received dinner (salmon or meatballs, Fjordland, Norway), cheese and Go`morgen yoghurt (Tine, Norway). Participants were responsible for the remaining ingredients of the diet plan (whole grain bread, butter, jam and fruits). In order to control for discrepancies between planned and actual intake, participants registered their food intake.

### Infusion and exercise protocol

After an overnight fast a cannula was inserted into a forearm vein in both arms.

A baseline blood sample was collected before participants received a standardized oatmeal breakfast (0.11 g protein •kg body mass-1), to be consumed within 20 minutes. Thirty minutes after the baseline blood sample a primed continuous infusion of [2H5]phenylalanine (0.05 μmol•kg-1•min-1; 2 μmol•kg-1 prime; Cambridge Isotopes Laboratories, Andover, MA, USA) was started. Biopsies and blood samples were collected according to Figure 1. The exercise session consisted of 4 sets of 8 repetitions to failure (8 RM) of leg press and knee extension, with a new set starting every 3 min. Warm-up sets of 10 repetitions at 50 and 80% of the 8RM loads were carried out in leg press.

### Supplements

Native whey and WPC-80 contained whey protein only, whereas milk contained 20% whey and 80% casein. Tine ASA (Oslo, Norway) produced the milk and whey supplements for this study. In order to match all drinks on macronutrients, cream (Tine, Norway), lactose (Arla food ingredients, Denmark), and water was added to WPC-80 and native whey (Table 2). Drinks were enriched with 6% [2H5] phenylalanine to avoid dilution plasma enrichment after intake. All drinks were matched for appearance and flavor.

**Table 2:** Amino acid and macronutrient content per serving of milk, WPC-80 and native whey

	**Milk (1% fat)**	**WPC-80**	**Native whey**
Alanine	0.6	1.0	1.1
Arginine	0.7	0.5	0.6
Aspartic acid	1.6	2.2	2.5
Cysteine	0.2	0.4	0.6
Phenylalanine	1.0	0.7	0.8
Glutamic acid	4.3	3.6	3.8
Glycine	0.4	0.4	0.4
Histidine	0.6	0.4	0.4
Isoleucine	1.0	1.3	1.2
Leucine	2.0	2.2	2.7
Lysine	1.7	1.9	2.3
Methionine	0.5	0.4	0.5
Proline	2.0	1.3	1.1
Serine	1.1	1.0	1.0
Threonine	0.9	1.5	1.1
Tyrosine	0.8	0.4	0.6
Valine	1.3	1.2	1.1
Tryptophan	0.3	0.3	0.5
Total protein	20.5	19.7	21.2
Fat	6.3	6.7	6.9
Carbohydrate	38.2	42.0	40.7

### Dual-energy X-ray absorptiometry

Body composition was assessed by dual energy X-ray absorptiometry (Lunar iDXA GE Healtcare, Madison, Wisconsin, USA, using the enCORE Software

Version 14.10.022) one week before the experiment. After refraining from exercise for 48 hours and an overnight fast, participants were scanned from head to toe in a supine position, providing values for lean tissue, fat mass and bone mineral content.

### Quadriceps force-generating capacity

In order to investigate whether the protein supplements affected the rate of recovery of muscle function, we assessed quadriceps function at 15 min, 5,5 and 24 hours after exercise. Quadriceps force-generating capacity was assessed as a isometric voluntary maximal contraction (MVC) in a custommade knee-extension apparatus (Gym2000, Geithus, Norway). Participants were seated with a four-point belt fixing the chest and hips to keep knee and hip joints at 90°. Three attempts of 3 s with 1 min rest between were given to reach MVC. Force was measured with a force transducer (HMB U2AC2, Darmstadt, Germany). MVC was tested after a 5 min warm up on a cycle ergometer, except for the measurement 10 minutes after the workout.

### Blood analyses

Serum samples were analyzed for creatine kinase and urea at Fürst Medical Laboratory (Oslo, Norway). Plasma insulin and glucose were measured using an enzyme-linked immune sorbent assay (Alpco, Salem, NH, USA) and a Cobas clinical analyzer (Cobas 6000, Roche Diagnostics, Indianapolis, IN, USA), respectively. Amino acid concentration was measured in plasma with an EZfaast amino acid analysis kit (Phenomenex®, Torrance, CA, USA) and gas chromatography/mass spectrometry (Shimadzu QP-2010 Ultra GCMS, Shimadzu Scientific Instruments, Columbia, MD) as described earlier (16).

### Biopsy collection and analyses

Muscle biopsies were collected from the mid portion of m. vastus lateralis with a modified Bergström technique with suction. Pre-analytical processing of muscle tissue was done as described earlier (19). Specimens were used to make a homogenate of soluble protein for analysis by Western Blot, and for analysis of mixed muscle FSR as a measure of MPS.

Samples for western blotting were treated as previously described (19), quantified with ChemiDoc MP (BioRad Laboratories CA, USA) and analyzed with Image Lab (v5.1, BioRad Laboratories CA, USA). Primary antibodies against p70S6K and phosphor-p70S6K Thr389 (1:1000 for both, cat. no. 8209), eEF-2 (1:5000, cat. no. 2332), phosphor-eEF-2 Thr56 (1:5000, cat. no. 2331), 4EBP-1 (1;1000, cat. no. 9452), phosphor-4EBP-1 Thr37/46 (1:1000, cat. no. 9455) and secondary antibody against anti-rabbit (1:3000, cat. no. 7074) were bought from Cell Signaling (Beverly, MA, USA), diluted in a 1% fat-free skimmed milk and 0.05% TBS-t solution. All samples were run in duplicates. Blood, muscle protein-bound and intracellular free phenylalanine enrichment was analyzed according to Wolfe and Chinkes (20) and Burd and colleagues (21), as described previously (22).

### Calculations

Baseline muscle fractional synthesis rate (FSR) was calculated using the precursor product method (20):

FSR (%h-1) = Ep2 –Ep1 /(Epre • t) • 100

The product is the difference in enrichment of the bound protein pool (Ep2) and the mixed plasma proteins (Ep1). The precursor (Epre) is the average plasma free or muscle free D5 phenylalanine enrichments to estimate the upper (muscle free) and lower (plasma free) limits of the true muscle protein FSR. The tracer incorporation time is denoted by t.

Skeletal muscle fractional synthesis rate (FSR) was calculated (as a measure of MPS) according to the precursor product method where the precursor is the mean enrichment of the intracellular pool (EIC) of biopsies being analyzed (20). The product is the difference in enrichment of the bound protein (EBP) pools of the two muscle biopsies being analyzed. Skeletal muscle FSR is expressed as percent per hour: FSR (%/hour) = ((EBPt 2-EBPt 1)/(EIC•(t 2-t 1)))•100

The baseline MPS was only calculated during the first experiment for participants in the whey group, and this value was used as a baseline for both supplements in this group.

### Statistics

Non-normally distributed data (D’Agostino and Pearson omnibus normality test) were log-transformed prior to statistical analysis. All data are illustrated in original form. Comparisons of WPC-80 with native whey were analyzed by one-way repeated measures ANOVA. Comparisons with milk were made by a two-way ANOVA with repeated measures (time x group). Tukey´s multiple comparisons test was used as a post hoc test to specify the significant differences between groups and time points. Comparisons between time points within groups were only made against the pre-value and a Dunnett´s multiple comparison test was used as a post hoc test. Subject characteristics and area under the curve differences between groups were analyzed with a one-way non-repeated measure ANOVA. Pearson’s correlation coefficient (r) was used to investigate relations between variables. Statistical power was calculated using a standard deviation of 0.04 %/h, giving us an 80% power to detect a true group mean difference of 0.024 %/h for the comparison between native whey and WPC-80, and 0.053 %/h for the comparison between native whey and milk with 10 participants in each group (StatMate, Graphpad Software, San Diego, CA, USA). Statistical analyses were made using Prism Software (Graphpad 6, San Diego, CA, USA). All results are expressed as means ± SD. Statistical significance level was set at p ≤ 0.05.

## Results

Participant characteristics were not significantly different between groups, but the participants in the whey group tended to be younger than the participants in the milk group (Table 1).

### Diet standardization and exercise variables

There were no differences between the groups in terms of caloric (milk: 29.8 kcal•kg-1•day-1, whey: 28.6 kcal•kg- 1•day-1, P = 0.75) and protein intake (milk: 1.3 g•kg-1•day-1, whey: 1.1 g•kg-1•day-1, P = 0.20) before or during the study.

No group differences were observed for training volume 8RM in leg press (P = 0.749), knee extensions (P = 0.740) and training volume (load x repetitions; P = 0.683) during the workout (Table 1).

### Blood glucose, insulin, urea and creatine kinase

Plasma glucose (Figure 2A) increased (P < 0.05) in response to both supplement servings and returned towards baseline within 60 min after ingestion in all groups. Plasma insulin (Figure 2B) increased in all groups after supplement ingestion and remained elevated until 300 min with all supplements (P < 0.02). CK levels (data not shown) were elevated in all groups 24 hours after exercise (P < 0.001). No group differences were observed for plasma glucose, insulin or serum CK.

Serum urea (data not shown) increased after ingestion of WPC-80 and native whey at 180 and 300 min (P < 0.01), and was higher with native whey than milk at 300 min (P = 0.043).

### Blood amino acid concentrations

**Figure 3:**
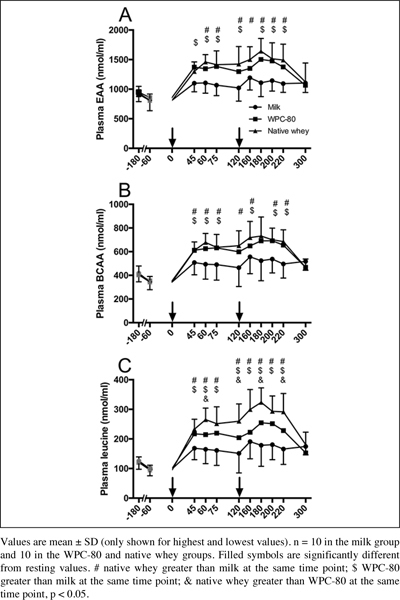
Blood concentrations of essential amino acids (A), branched chained amino acids (B) and leucine (C) following intake of milk, WPC-80 and native whey after a bout of heavy leg resistance exercise in elderly individuals. Arrows indicate timepoints of protein supplement ingestion

All supplements increased blood concentrations of total amino acids, EAA, total BCAA and individual BCAAs (P < 0.01; Figure 3). Native whey increased blood leucine concentrations more than WPC-80 at 65, 120, 180 and 220 min after protein intake (P < 0.05). Native whey and WPC-80 increased blood concentrations of leucine, BCAA and EAA, but not valine and total amino acids, to a greater extent than milk (P < 0.05). Leucine area under the curve was greater with native whey than WPC-80 and milk (45%, P = 0.014 and 130%, < 0.001, respectively), and greater with WPC-80 than with milk (60%, P = 0.036).

### Signaling

**Figure 4:**
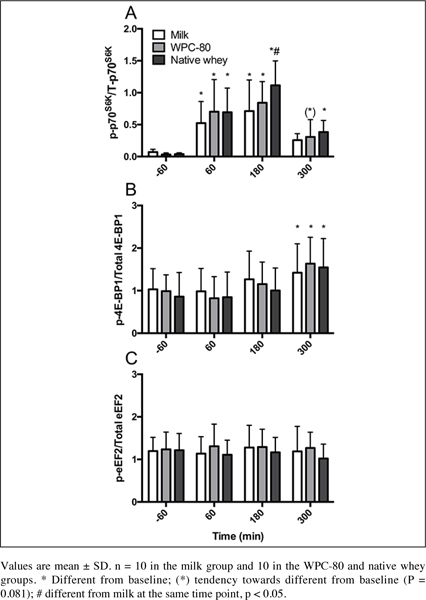
Ratio between phosphorylated and total P70S6K (A), (B) 4E-BP1 and eEF-2 (C) following intake of milk, WPC-80 and native whey after a bout of heavy leg resistance exercise in elderly individuals

Phosphorylation of P70S6K increased in all groups at 60 and 180 min post exercise time points (P < 0.003; Figure 4). Native whey had greater phosphorylation of P70S6K than milk at 180 min (P = 0.014) and was still increased compared to baseline at 300 min (P = 0.019). Phosphorylation of 4EBP-1 was increased from baseline (milk: P = 0.025, WPC-80 and native whey: P < 0.001). We did not observe any significant changes from baseline foreEF-2. No group differences were observed for 4E-BP1 or eEF-2.

### Muscle protein synthesis

**Figure 5:**
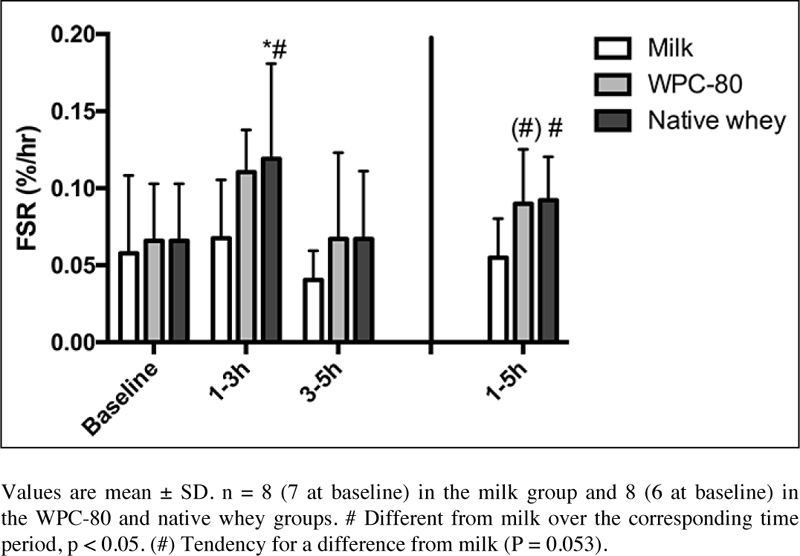
Mixed muscle FSR values following intake of milk, WPC- 80 and native whey during a 5-hour period after a bout of resistance exercise

We observed no differences in MPS between WPC-80 and native whey (Figure 5). In the early period (1-3 hours) native whey was significantly higher than baseline (P = 0.004) and reached higher rates of MPS than milk (P = 0.041). There were no differences for MPS within or between groups during the late period (3-5 hours). For the total period MPS values tended to be higher with WPC-80 and was higher with native whey than in milk (P = 0.053 and 0.044, respectively). [2Hs] phenylalanine TTR in blood did not differ significantly between groups, but was significantly increased between 175 and 190 min with all supplements (data not shown).

### Recovery of muscle function

All groups displayed a drop in force-generating capacity after the workout (native whey: -16.8 ± 4.0%, WPC-80: -17.8 ± 8.1%, milk: -12.7 ± 2.6%; P < 0.001; data not shown). At 24 hours, milk and WPC-80 was still significantly different from baseline values, whereas native whey was not. There were no differences between groups.

### Correlations

Blood leucine concentration at 45 min, blood leucine peak and area under the curve correlated with P70S6K phosphorylation at 180 min (r = 0.40-0.54, P < 0.05).

## Discussion

The present study tested the hypothesis that native whey would have a greater acute anabolic effect on muscle than WPC-80, when supplemented as 20 g protein doses immediately and two hours after resistance exercise in elderly individuals. We observed no differences between WPC- 80 and native whey in the ability to stimulate post exercise phosphorylation of P70S6K, 4E-BP1 and eEF-2 or rates of MPS.

All supplements increased blood concentrations of amino acids, native whey more so than WPC-80 for leucine, and the whey supplements more than milk for EAA, BCAA and leucine. The observed changes in blood amino acids concentrations mirrored the results from previous studies comparing whey proteins to casein (23) and milk (16). The differences in blood concentrations of EAA and leucine between the whey supplements and milk were smaller, and reached lower peak values after the first serving in our elderly participants than previously observed in young men (16). This could be related to altered digestion, absorption or a greater first pass splanchnic extraction of protein in elderly (24). Still, the combination of resistance exercise and supplementation was able to robustly increase phosphorylation of P70S6K in the elderly participants reflecting the differences in blood concentrations of leucine, BCAA and EAA on a group level.

Phosphorylation of p70S6K was elevated with all supplements after exercise (Figure 4A), and displayed the same temporal pattern as previous studies supplementing with milk protein or EAA after resistance exercise in elderly individuals (11, 12). We observed a greater phosphorylation of p70S6K with native whey than milk and moderate positive correlations between blood concentrations of leucine and p70S6K phosphorylation. These data supports the central role of leucine in stimulating MPS (25). Although we observed p70S6K phosphorylation and the MPS response to be reflective of the leucine concentration in blood during the early period, there were no correlations between MPS and leucine concentrations in blood or p70S6K phosphorylation. Several factors might explain this: 1) Regulation of MPS is complex and involves several pathways and kinases. Investigating a few of these is likely to give an incomplete picture (26), 2) The relationship between p70S6K and MPS is not necessarily linear (27), 3) A factual correlation between the “snap-shot” nature of the biopsy for western blotting and the prolonged measurement of MPS may easily be missed (26).

Phosphorylation of 4E-BP1 was delayed relative to the peak MPS response (Figure 4B). Still, the time course of 4E-BP1- phosphorylation is similar to that reported in previous studies investigating protein ingestion and resistance exercise in young (28, 29) and elderly (30). In contrast to previous studies in elderly (30) we did not observe a decreased phosphorylation of eEF-2 in response to resistance exercise and protein ingestion (Figure 4C). However, studies in young have reported no change in eEF-2 phosphorylation in response to resistance exercise and protein ingestion (31). Being substrates of mTORC1, phosphorylation of p70S6K, 4E-BP1 and eEF-2 are expected to correspond. However, differences in the timing of signaling events between these kinases, input from other pathways and sensitivity towards different anabolic stimuli may complicate this picture. In contrast to p70S6K, 4E-BP1 does not respond to rapamycin treatment (32), which is thought to inhibit the growth factor and amino acid response of mTORC1 (33). Thus, 4E-BP1 may not be as responsive to leucine as p70S6K, as our results would suggest.

Previous studies have shown that in contrast to young participants (34) ingestion of 20 g whey protein seem to be suboptimal for maximal stimulation of MPS in elderly, both at rest and after unilateral leg resistance exercise (5). Further, adding 4.5 g of leucine to a suboptimal dose of protein (11, 35) or 2.5 g of leucine to 20 g of casein (36) enhanced the MPS-response in elderly. In the current study we observed no difference in post exercise MPS with native whey compared to WPC-80 in elderly participants. This may relate to the difference in leucine content between the supplements. In the current study, native whey contained only 0.6 g leucine more than WPC-80 per serving (1.2 g in total), which is considerably less than in previous studies (11, 36). Alternatively, a saturation of the MPS-response could lead to similar results with WPC-80 and native whey. In light of previous studies showing a dose response to whey protein in elderly at least up to 40 g after exercise (37), we believe this to be unlikely. Thus, this study suggests that the dose of leucine added to a suboptimal dose of whey protein should be greater than 0.6 g per serving in order to have an effect on MPS in elderly. A small increase in the difference in leucine content between milk and native whey (0.86 g per serving) did lead to a difference in MPS-response. However, this difference may also be the result, at least in part, of other factors such as rate of absorption (28, 38).

WPC-80 did not increase significantly from baseline in the early period. However, based on the values obtained and previous studies supplementing with whey protein in elderly (23, 37) this seems to be a matter of low statistical power.

Native whey reached higher MPS rates than milk during the early (1-3h), and total period (1-5h). This is in agreement with previous studies in young (39) and elderly (23) individuals comparing casein (making up 80% of protein in milk) and whey supplementation after resistance exercise. Thus, ingestion of native whey protein in combination with resistance training may be advantageous compared to casein or milk in terms of combating sarcopenia. Long-term studies are needed to answer this hypothesis.

Although milk is considered a high quality protein source, we observed no stimulatory effect of milk on post exercise MPS in elderly participants. This may relate to anabolic resistance in our elderly participants. However, similar responses have been reported in response to 22 g of micellar casein, in young individuals (39). The concept of anabolic resistance to protein intake and resistance exercise in elderly is complex, and in need of further investigation (5). It has been hypothesized that the anabolic resistance in elderly primarily manifests itself only when a suboptimal stimulus is applied (5). Thus, young should respond to lower training volumes (40) and doses of protein than elderly (7). On the contrary, if the stimuli are strong enough or combined, elderly would reach the same MPS responses as young (11, 41). In the current study, we applied a relatively large training volume for the legs and 2 x 20 g of high quality protein in all groups. However, no significant effects were observed on MPS for milk or WPC-80 in the early period, or any of the supplements in the late period. We hypothesize that combining the two suboptimal 20 g servings into one potentially optimal serving of 40 g of protein would have elicited a greater effect on MPS over the total period. The standardized breakfast may also have contributed to slightly elevated MPS-rates in the baseline period, thus making it harder to observe a difference between baseline and post exercise periods.

We observed an 8-35% reduction in force-generating capacity measured 10 min after the workout. Together with the small increases in blood CK levels, this suggests mild to moderate muscular stress (42). The small differences in MPS during the first hours after exercise did not lead to a measurable difference in recovery of force-generating capacity at 24 hours after exercise.

We were unfortunately not able to measure MPB in this study. Earlier studies in young individuals have found MPS to respond with greater changes than MPB (43-45). We therefore assume that our MPS measurements reflect the major part of the net protein balance response to protein supplements after resistance exercise. There was a larger part of women in the native whey group, but we did not observe any significant sex differences for the measured variables. As our participants were healthy and active our results are not necessarily representative of the sedate and less healthy group of elderly, a group in which the need for interventions might be greater.

Future studies should investigate if the acute differences observed between native whey and milk are evident during a training and supplementation intervention, and whether functional capacity can be improved by supplementation.

## Conclusions

Despite an apparently favorable increase in blood leucine concentrations after ingestion of native whey protein, there were no significant differences between native whey and WPC- 80 in stimulating the phosphorylation p70S6K, eEF-2 and 4E-BP1 or MPS during a 5-hour post exercise period in elderly individuals. Thus, in order to further stimulate MPS by adding leucine to a suboptimal dose of whey protein in elderly, more than 0.6 g of leucine is needed.

*Acknowledgements:* The authors would like to thank Hege Østgaard, Eugenia Carvalho for excellent lab work. The participants are acknowledged for their valuable contributions.

*Conflict of interest:* Håvard Hamarsland, Anne Lene Nordengen, Sigve Nyvik Aas, Kristin Holte, Ina Garthe, Gøran Paulsen, Matthew Cotter, Elisabet Børsheim, Haakon B. Benestad and Truls Raastad declare no conflict of interest.

*Founding:* TINE SA and the Norwegian Research Council funded the study (NRC). TINE SA and NRC was not involved in design of the study, data collection, analyses, interpretation of data or writing of the manuscript. EB and MC are partially supported by USDA-ARS-6026-51000-010-05S, Arkansas Biosciences Institute (the major research component of the Arkansas Tobacco Settlement Proceeds Act of 2000), NIH-AG033761 and NIH-P30AG028718.

*Ethical standards:* The study was approved by the Regional Ethics Committee for Medical and Health Research of South-East Norway (2014/834/REK sørøst C) and performed in accordance with the Declaration of Helsinki. All participants signed a written informed consent form before entering the study. The trial was registered at clinicaltrials. gov as NCT03033953.

## Electronic supplementary material

**Supplementary figure 1**
Participant flowchart

**Supplementary figure 2** Blood concentrations of essential amino acids (except leucine) following intake of milk, WPC-80 and native whey after a bout of resistance exercise. Arrows indicate time-points of protein supplement ingestion. Values are mean ± SD (only shown for highest and low values). n = 10 in the milk group and 10 in the WPC-80 and native whey groups. Black symbols are significantly higher than resting values. # native whey greater than milk at the same time point; $ WPC-80 greater than milk at the same time point; & native whey greater than WPC-80 at the same time point, p < 0.05.

**Supplementary figure 3** Blood concentrations of non-essential amino acids following intake of 20 g milk protein, WPC-80 and native whey after a bout of resistance exercise. Arrows indicate time-points of protein supplement ingestion. Values are mean ± SD (only shown for highest and lowest values). n = 10 in the milk group and 10 in the WPC-80 and native whey groups. Black symbols are significantly higher than resting values. # native whey greater than milk at the same time point; $ WPC-80 greater than milk at the same time point; & native whey greater than WPC-80 at the same time point, p < 0.05.
