# Supplementary figures and images for: Native Whey Induces Similar Post Exercise Muscle Anabolic Responses as Regular Whey, Despite Greater Leucinemia, in Elderly Individuals
Source: J Nutr Health Aging. 2018 Sep 18;23(1):42–50. doi: 10.1007/s12603-018-1105-6 (PMC6332708; doi:10.1007/s12603-018-1105-6)

**Supplementary figure 1**

Participant flowchart


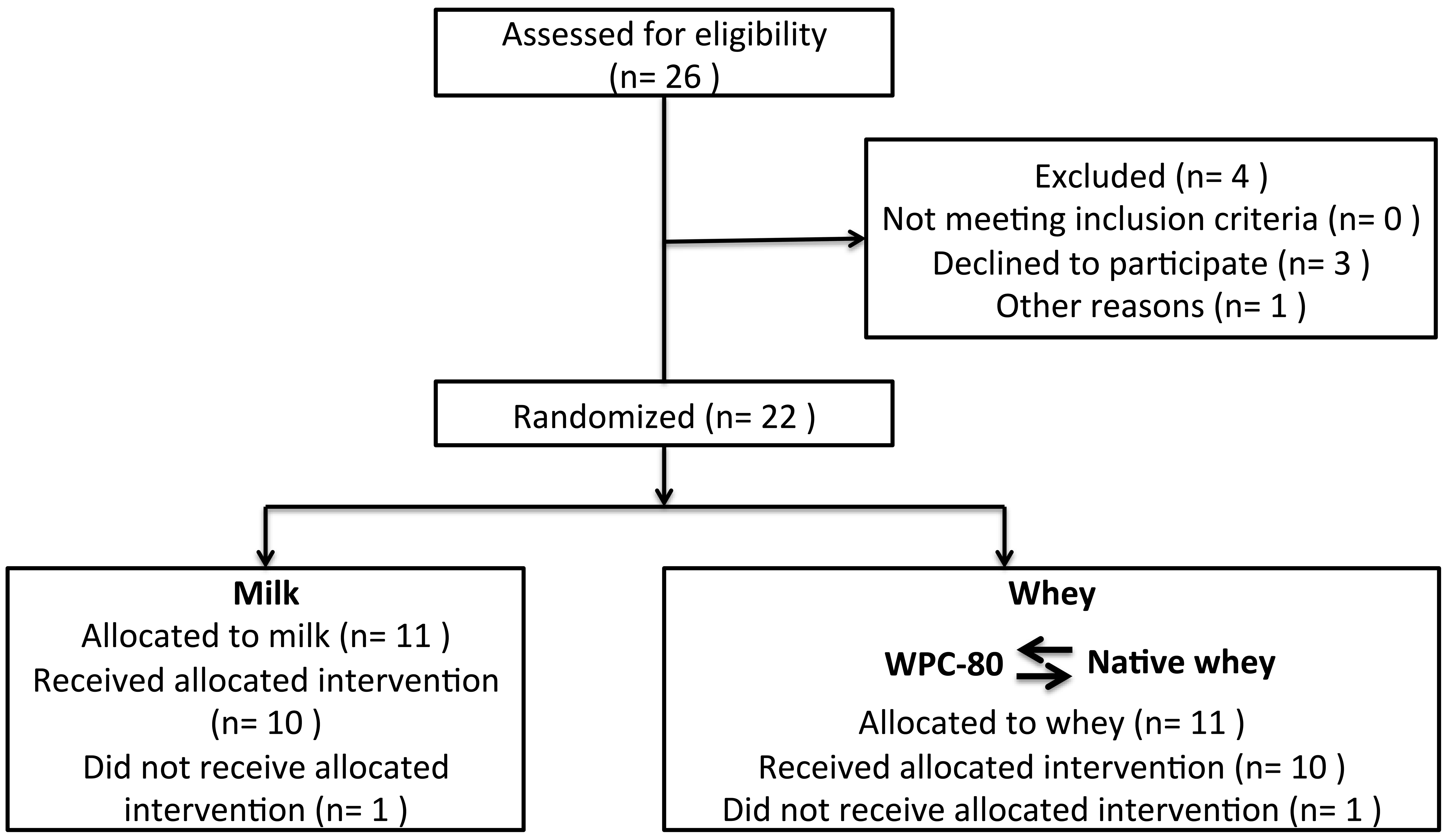

Supplement: Supplementary file 1 — Supplementary figure 1 Participant flowchart [file mmc1.docx]
