# Supplementary material for: Native Whey Induces Similar Post Exercise Muscle Anabolic Responses as Regular Whey, Despite Greater Leucinemia, in Elderly Individuals
Source: J Nutr Health Aging. 2018 Sep 18;23(1):42–50. doi: 10.1007/s12603-018-1105-6 (PMC6332708; doi:10.1007/s12603-018-1105-6)

**Supplementary figure 2** Blood concentrations of essential amino acids (except leucine) following intake of milk, WPC-80 and native whey after a bout of resistance exercise. Arrows indicate time-points of protein supplement ingestion. Values are mean ± SD (only shown for highest and low values). n = 10 in the milk group and 10 in the WPC-80 and native whey groups. Black symbols are significantly higher than resting values. # native whey greater than milk at the same time point; $ WPC-80 greater than milk at the same time point; & native whey greater than WPC-80 at the same time point, p < 0.05.


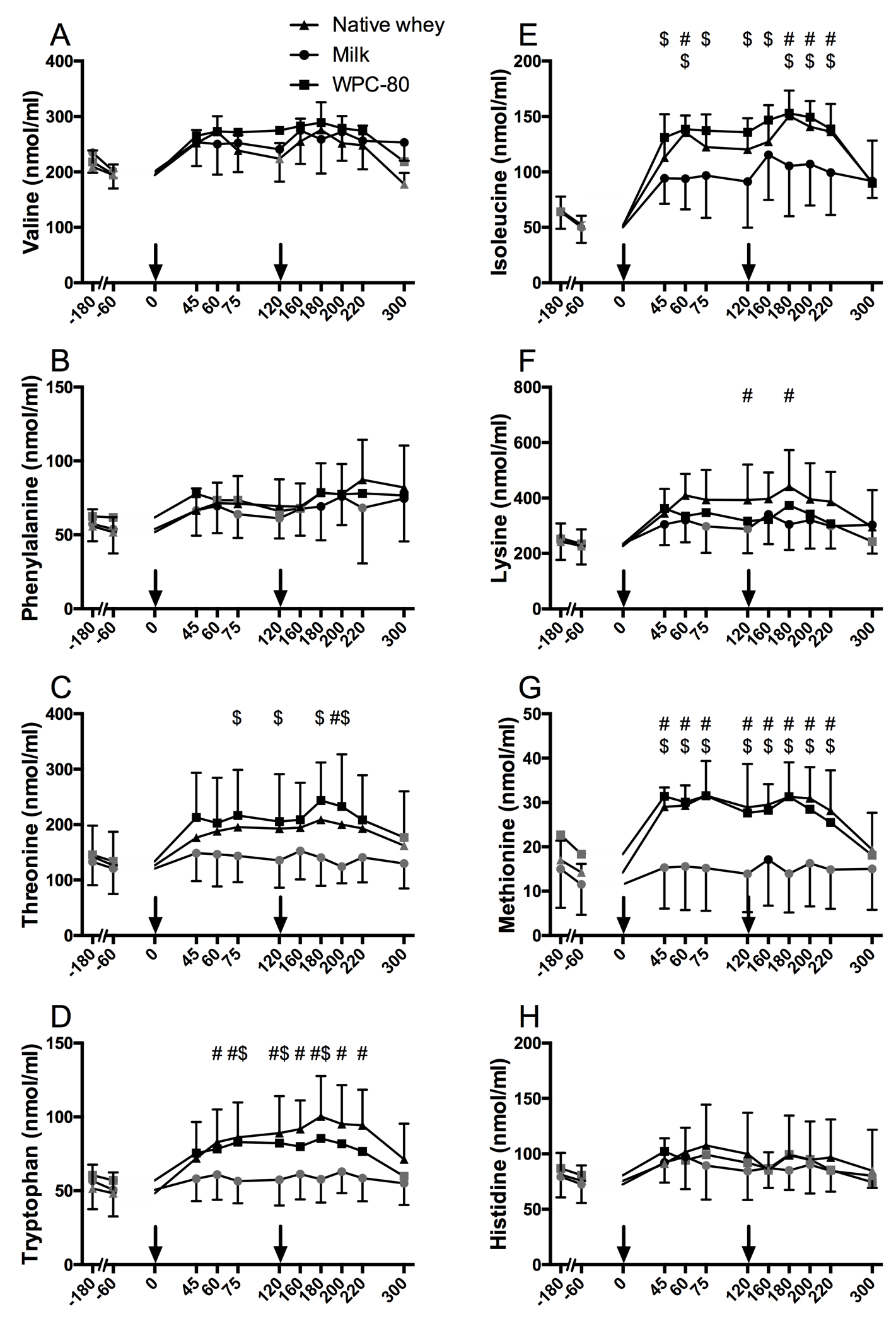

Supplement: Supplementary file 2 — Supplementary figure 2 Blood concentrations of essential amino acids (except leucine) following intake of milk, WPC-80 and native whey after a bout of resistance exercise. Arrows indicate time-points of protein supplement ingestion. Values are mean ± SD (only shown for highest and low values). n = 10 in the milk group and 10 in the WPC-80 and native whey groups. Black symbols are significantly higher than resting values. # native whey greater than milk at the same time point; $ WPC-80 greater than milk at the same time point; & native whey greater than WPC-80 at the same time point, p < 0.05. [file mmc2.docx]
